# High Prevalence of Tuberculosis in Previously Treated Patients, Cape Town, South Africa

**DOI:** 10.3201/eid1308.051327

**Published:** 2007-08

**Authors:** Saskia den Boon, Schalk W.P. van Lill, Martien W. Borgdorff, Donald A. Enarson, Suzanne Verver, Eric D. Bateman, Elvis Irusen, Carl J. Lombard, Neil W. White, Christine de Villiers, Nulda Beyers

**Affiliations:** * Stellenbosch University, Cape Town, South Africa; † Academic Medical Center, Amsterdam, the Netherlands; ‡ KNCV Tuberculosis Foundation, The Hague, the Netherlands; § International Union Against Tuberculosis and Lung Diseases, Paris, France; ¶ University of Cape Town, Cape Town, South Africa; # Medical Research Council, Cape Town, South Africa; ** City of Cape Town Health Department, Cape Town, South Africa

**Keywords:** Survey, recurrence, tuberculosis, prevalence, South Africa, research

## Abstract

The tuberculosis (TB) notification rate is high and increasing in 2 communities in Cape Town, South Africa. In 2002, we conducted a prevalence survey among adults >15 years of age to determine the TB prevalence rate; 15% of households in these communities were randomly sampled. All persons living in sampled households were eligible for chest radiography and sputum examination. Of the 3,483 adults who completed a questionnaire, 2,608 underwent chest radiography and sputum examination. We detected 26 bacteriologically confirmed TB cases and a prevalence of 10.0/1,000 (95% confidence interval [CI] 6.2–13.8 per 1,000). We found 18 patients with smear-positive TB, of whom 8 were new patients (3.1/1,000, 95% CI 0.9–5.1/1,000). More than half of patients with smear-positive TB (10, 56%) had previously been treated. Such patients may contribute to transmission of *Mycobacterium tuberculosis* and the high TB prevalence rate. Successful treatment of TB patients must be a priority.

## Abstract 2

More than half of smear-positive case-patients had previously undergone treatment.

In 2003, South Africa had an estimated incidence of 218 new smear-positive tuberculosis (TB) cases per 100,000 population. This country ranked eighth in the world for total number of TB cases per country and tenth for incidence rates (*1*). Western Cape Province had the highest notification rate in this country (*2*). In Cape Town, the largest city in this province, the notification rate for new smear-positive TB was 266/100,000 in 2002 (*3*). In Ravensmead and Uitsig, 2 neighboring urban communities in Cape Town, the rate of registered new smear-positive TB cases increased from 228/100,000 in 1994 to 299/100,000 in 1998 and to 341/100,000 in 2002 (*4*,*5*).

Although improved case detection may account for these increases, a true increase in incidence may also be occurring. Currently, there is no reliable estimate of the TB situation in this region. Furthermore, no data are available on number of undetected cases in the 2 communities, care-seeking behavior and delay in diagnosis, and number of persons who previously had TB, all of which are factors that may contribute to transmission of TB. We suspect that this population contains a substantial number of TB patients who previously had this disease, and that this group contributes to transmission of TB and the persistent high rates of TB notification.

The aim of this TB prevalence survey was to obtain a reliable estimate of the situation, and to determine the number of undetected cases in the 2 communities, the number of persons who have previously had TB, and the proportion of previously treated cases among undetected TB patients. Results of this study will be used to plan interventions to control the TB epidemic in these 2 communities in Cape Town.

## Methods

### Study Area

We conducted a TB prevalence survey in the communities of Ravensmead and Uitsig, which have a total surface area of 3.5 km^2^. In 2001, the study area had a population of 36,334 (*5*). Two primary healthcare clinics and an adjacent tertiary care hospital serve the area. The World Health Organization directly observed treatment short-course strategy was introduced in these 2 communities in 1997.

### Study Design

The TB prevalence survey was part of a larger community survey, the Lung Health Survey. In addition to prevalence of TB, the Lung Health Survey aimed to determine the prevalence of lung diseases, including asthma and chronic obstructive pulmonary disease. The current report deals only with TB. Information on other lung diseases will be reported elsewhere.

The study included 2 neighborhoods with similar socioeconomic status. We selected a random sample of all addresses in the study area and defined an address as the residential geographic location (either a physical street address or the name and number of a flat). A randomized 15% sample was taken from all residential addresses. A total of 5,592 addresses were situated in the study area, of which 839 households were selected for participation in the survey. The study protocol was reviewed and approved by the ethics committees of Stellenbosch University and the University of Cape Town.

### Survey Procedures

The Lung Health Survey was conducted from July 1 through December 15, 2002. Trained community workers counted the number of persons at each selected address. All persons at selected addresses (including the main house and all backyard shacks) were eligible for investigation. If the head of the household did not give consent for the household to be enrolled in the survey, the household was replaced with a household at an adjacent address, e.g., first to the right and then to the left of the household that declined participation. All persons living at the address were informed of the purpose of the survey, and written informed consent was obtained from each participant before enrollment. The Lung Health Survey included both adults and children, but here we report data in our survey only for adults >15 years of age.

All participants, supervised by a trained field worker, completed a questionnaire containing questions on demographic characteristics and earlier TB treatment. Patient category and treatment outcome of previous disease episode(s) were obtained from the local healthcare center.

Participants were then transported to a nearby facility where chest radiographs (35 × 43 cm postero-anterior view) were performed by using a 200-mA chest radiography machine. Within 1 week, a pulmonologist screened the radiographs for abnormalities. Persons with abnormalities that required immediate investigation or treatment were referred to a local hospital. An accredited reader assessed all radiographs when the survey was finished by using a standardized classification system developed for epidemiologic surveys (*6*). Abnormalities were classified as being either consistent with TB or not related to TB. Parenchymal, pleural, and central structure abnormalities were considered consistent with TB. A second experienced reader then reread a stratified sample of 31% of the radiographs. Kappa agreement between the 2 readers was 0.69 (95% confidence interval [CI] 0.64–0.74) for abnormalities consistent with TB and 0.47 (95% CI 0.42–0.53) for whether the radiographic result was normal (*6*). On the basis of these results, a second reader was deemed unnecessary.

Upon request, each participant provided a sputum specimen at the health center where the chest radiograph was obtained by using the active cycle of breathing technique (*7*). Specimens were collected in a wide-mouthed plastic container with a secure screw-top lid. This container was then transported to the laboratory where it was processed within 3 days. A second specimen was obtained from persons who had a 1 positive smear, a 1 positive culture, or both, before they were referred for treatment to a nearby clinic.

### Laboratory Procedures

One smear and 1 culture were prepared from each sputum specimen. Smears were stained by using the Ziehl-Nielsen technique with carbol fuchsin and methylene blue. The smears were scored by 1 reader according to the guidelines of the International Union Against Tuberculosis and Lung Disease (*8*). A smear result was considered positive if we observed >1 acid-fast bacillus per 100 oil-immersion fields. This provision included scanty smears because these are considered indicative of true positivity (*9*). Sputum samples were liquefied and decontaminated with 4% NaOH by using standard procedures (*10*). Samples were then centrifuged at 3,000 × *g* for 15 minutes. Concentrated decontaminated sputum sediment was resuspended in 2 mL phosphate buffer (68 mmol/L, pH 6.8) and placed on Löwenstein-Jensen slants. These slants were then incubated at 37°C for 6 weeks.

### Definitions, Data Processing, and Statistical Analysis

A bacteriologically confirmed case of TB was defined as a person with either 2 positive smears or 2 positive cultures, or 1 positive smear and 1 positive culture. Participants with 1 positive smear or 1 positive culture whose chest radiographs showed TB-related abnormalities, or those with a positive sputum smear or culture results from specimens collected at the health center within 2 months after sputum collection in the prevalence survey, were also considered to have bacteriologically confirmed TB.

Data were entered into a Microsoft (Redmond, WA, USA) Access database. Inconsistencies between the 2 entries were checked against original data and errors were corrected. Statistical analysis was performed by using SPSS version 12.0.1 for Windows (SPSS Inc., Chicago, IL, USA) and STATA version 8.0 for Windows (*11*). Prevalence of TB was calculated by dividing the number of TB cases by the number of participants who attempted to provide a sputum specimen. Those who were unable to provide a sputum specimen were considered smear-negative and culture-negative. Results include crude prevalence rates and prevalence rates adjusted for the cluster sampling design. Households served as the primary sampling units and variation at this level was taken into account.

We calculated prevalence after applying a correction factor for 100% collection coverage. Results were weighted to adjust for unequal coverage in the different sex and age groups (15–34 and >35 years of age). Separate weighting was applied to participants in the original and replacement samples. We calculated the sampling weight as the product of (1/selection probability of a household) and (1/participation rate). The selection probability of a household was 0.15 because we randomly sampled 15% of the households. The participation rate varied by group sampled; e.g., 68% of women 15–34 years of age in the original sample had a chest radiograph performed. Sampling weight was (1/0.15) × (1/0.68) = 9.8. CIs were calculated by using the normal approximation to the binomial distribution.

## Results

### Study Population and Coverage of Measurements

Initially, heads of 625 (74%) of 839 households consented to participate in the study. We replaced 212 of 214 households that refused participation; 2 households could not be replaced. Of 214 nonparticipating households, 81 provided demographic details of persons living in the household. Occupants of these 81 households did not differ from the 212 households that were resampled with respect to sex (odds ratio [OR] 1.00, 95% CI 0.82–1.23) or age (t = 0.33, p = 0.74, by Student *t* test). In the final sample, residents within 837 households included 3,971 adults, of whom 3,483 (88%) consented and completed a questionnaire (Figure). Of these, 2,608 (75%) had a chest radiograph and attempted to provide a sputum specimen. Persons >35 years of age (78%) had a chest radiograph more often than persons <35 years of age (72%) (OR 1.41, 95% CI 1.21–1.65). More women (78%) than men (71%) had a chest radiograph (OR 1.48, 95% CI 1.26–1.72). Of those who had a chest radiograph, 1,170 (45%) were able to provide a sputum specimen. Thirteen specimens were contaminated or inadequate for culture because of insufficient volume.

**Figure:**
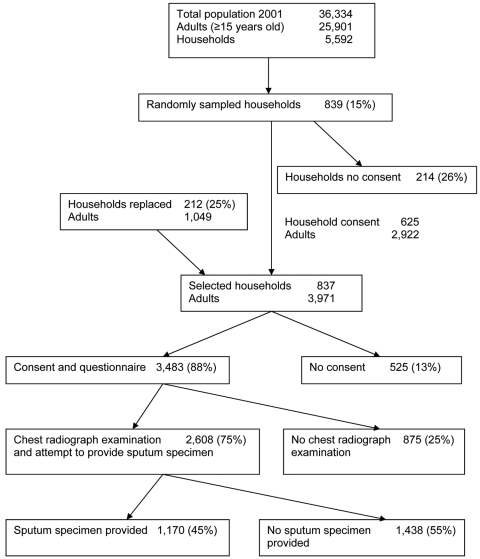
Sample selection of 3,483 adults, Cape Town, South Africa, for the study.

### Prevalence of TB

Of 2,608 participants with chest radiographs, 702 (26.9%) had an abnormality on the radiograph (95% CI 25.2%–28.6%). In 337 (12.9%) of these radiographs, abnormalities were consistent with TB (95% CI 11.6%–14.2%) (Table 1). Prevalence of TB-related abnormalities on chest radiographs increased with age (χ^2^ for linear trend 59.1, p<0.001) and were more often seen in men (16%) than in women (11%) (OR 1.48, 95% CI 1.16–1.89).

**Table 1:** Prevalence estimates of tuberculosis (TB) in adults ≥15 years of age, Cape Town, South Africa*

Characteristic	No. positive/ no. tested	Crude prevalence estimate	Prevalence estimate corrected for simple random sampling at household level	Weighted prevalence estimate, corrected for sampling and nonresponse
History of TB (questionnaire)	338/3,483	9.7% (8.7%–10.7%)	9.7% (8.6%–10.8%)	9.8% (8.7%–10.9%)
TB abnormalities on chest radiograph	337/2,608	12.9% (11.6%–14.2%)	12.9% (11.6%–14.3%)	12.9% (11.5%–14.3%)
Total smear-confirmed TB	18/2,608	6.9 (3.7–10.1)	6.9 (3.3–10.5)	7.1 (3.3–10.8)
New smear-confirmed TB	8/2,608	3.1 (0.9–5.2)	3.1 (0.9–5.2)	3.0 (0.9–5.1)
Total bacteriologically confirmed TB	26/2,608	10.0 (6.2–13.8)	10.0 (5.7–14.3)	10.2 (5.8–14.6)
New bacteriologically confirmed TB	16/2,608	6.1 (3.1–9.1)	6.1 (3.0–9.3)	6.2 (3.0–9.3)

*Prevalence estimates are per 1,000 unless otherwise stated (percentage values). Values in parentheses are 95% confidence intervals.

Twenty-nine participants had a positive smear or positive culture, of whom 3 did not fulfill the definition of a bacteriologically confirmed TB case; 2 had a scanty smear and 1 had a positive culture. The remaining 26 participants fulfilled the definition of bacteriologically confirmed TB, giving a prevalence of 10.0/1,000 (95% CI 6.2–13.8/1,000) (Table 1). Of these 26 patients with bacteriologically confirmed TB, 16 (62%) were new patients (no previous treatment), giving a prevalence of new bacteriologically confirmed TB cases of 6.1/1,000 (95% CI 3.1–9.1/1,000). Ten (56%) of the 18 smear-positive patients had been previously treated for TB. The prevalence of previously treated smear-positive TB cases was 3.8/1,000 (95% CI 1.5–6.2/1,000). Only 8 (44%) smear-positive cases were new cases, and the prevalence of new smear-positive TB cases was 3.1/1,000 (95% CI 0.9–5.1/1,000).

Correction for the cluster sampling design and adjustment for noncoverage had little effect on the prevalence estimates (Table 1). Three of the 26 patients with bacteriologically confirmed TB were receiving anti-TB therapy at the time of the survey. Nineteen of the remaining 23 patients with detected cases began treatment <6 months after these cases were detected in the survey. Of those patients, 12 were cured, 1 completed treatment without confirmed smear conversion, 2 interrupted treatment, 1 died, and 3 had an unknown outcome.

### History of TB

All 10 patients with bacteriologically confirmed TB who had previously received treatment for TB were smear-positive. Four of the 10 previously treated patients had been considered cured during their previous disease episodes, and 3 had interrupted treatment before completion. For 3 previously treated smear-positive patients, no information was available on the outcome of their previous disease episode (Table 2). Four patients had been treated for TB on more than 1 occasion before their cases were detected in the survey. Also, 3 previously treated patients lived in the same household. Bacteria isolated from 1 of 7 previously treated TB patients who underwent drug sensitivity testing were resistant to isoniazid (Table 2).

**Table 2:** Treatment history of 10 previously treated tuberculosis (TB) patients with sputum smear–positive TB test results, Cape Town, South Africa*

Patient no.	Patient category	Year of previous episode	Outcome	Drug resistance
1	New	1985	Cured	NT
	Retreatment after cure	1987	Cured	NT
	Retreatment after cure	1994	Cured	Sensitive
	Retreatment after cure	2000	Cured	Sensitive
	Retreatment after cure	2002	Cured	Sensitive
2	New	1996	Cured	Sensitive (2002)
3	New	1999	Cured	Sensitive (2002)
4	New	2001	Cured	Sensitive (2002)
5	New	1995	Interrupted	NT
6	Retreatment	1995	Interrupted	Sensitive
	Retreatment after interruption	1998	Failed	Sensitive
7	Retreatment	1997	Interrupted	INH resistant (2002)
8	Retreatment	1997	Unknown	NT
9	Unknown	Unknown	Unknown	NT
10	Unknown	Unknown	Unknown	Sensitive (2002)

*NT, not tested; INH, isoniazid.

A history of TB was reported by 338 (9.7%) of 3,483 adults (95% CI 8.7%–10.7%) (Table 1). The prevalence of bacteriologically confirmed TB was 29.9/1,000 (95% CI 11.4–47.7/1,000) in those who were previously treated for TB compared with 5.1/1,000 (95% CI 2.6–7.6/1,000) for persons who had never had TB (OR 5.96, 95% CI 2.68–13.25). Participants reporting a history of TB were significantly older (mean 41 years) than participants who had never had TB (mean 38 years) (t = 2.59, p = 0.01, by Student t test). A history of TB was more common in men (12%) than in women (8%) (OR 1.60, 95% CI 1.27–2.01).

## Discussion

The prevalence of bacteriologically confirmed TB in our study was high, and the proportion of patients that had >1 previous episode of TB was substantial. These data support the hypothesis that previously treated cases form a large proportion of TB patients in the 2 communities. Therefore, persons with previously treated TB are likely to contribute to transmission of *Mycobacterium tuberculosis*, and may also be a factor in maintaining high prevalence rates of TB.

Low treatment success rates may partly explain the high proportion of previously treated cases. Treatment success rates for new smear-positive cases were 78% in 2001 and 80% in 2002 (*4*), which were lower than the 85% recommended by the World Health Organization (*1*). Low cure rates in TB patients may lead to reactivation of this disease and could also result in patients with chronic TB who excrete TB bacilli over an extended period. However, recent studies using DNA fingerprinting showed that a large proportion of recurrent TB in our study area is caused by exogenous reinfection rather than reactivation (*12*,*13*). Approximately 10% of the survey population had previously had TB, as shown by responses to the question of whether persons had ever had TB and by TB-related abnormalities on chest radiographs. Previously treated persons had a much higher prevalence rate of TB than persons who had never had TB. Achieving high cure rates and preventing TB patients from infecting other people must therefore be considered the highest priority.

The high proportion of previously treated undetected cases suggests that case detection for previously treated cases is insufficient. A case-detection rate of 118% was reported for new smear-positive cases in South Africa (*1*), but no information on previously treated cases has been reported. Further research is needed to determine the case-detection rate for previously treated patients.

Previously treated TB is associated with multidrug-resistant TB and may be associated with extensively drug-resistant TB. A survey on drug resistance conducted in 1992–1993 in the Western Cape Province showed a rate of 8.6% acquired and 3.2% initial drug resistance in Cape Town (*14*). Recently, a study among hospitalized children (<13 years of age) showed an increase in isoniazid resistance from 6.9% in 1994–1998 to 12.8% in 2003–2005 (*15*,*16*). Drug-resistant TB is probably not the driving force behind the high prevalence of TB in the 2 study communities; however, it is likely to become an increasingly important factor.

Similarly, an increasing prevalence of HIV may be partly responsible for the increasing notification rates for TB, but does not adequately explain the high prevalence of TB in this study (*17*). HIV prevalence in the study area is not known and was not measured in this study. However, we believe that the prevalence rate for HIV in the study area is less than the rate of 12.4% in women attending public antenatal clinics in Cape Town in 2002 (*18*). Approximately 6% of patients with newly diagnosed TB in Ravensmead and Uitsig are HIV positive.

The high prevalence rates of TB in Ravensmead and Uitsig are reminiscent of similar observations in Inuit communities in Greenland, Canada, and the United States. In the Inuit population, an average annual incidence of active TB of 1,310/100,000 was reported from 1967 through 1969 (*19*). The highest rates of TB were reported in persons who had previously had TB (*20*), which was also observed in our study.

Our study was limited by a sample size that was insufficient to give precise prevalence estimates for bacteriologically confirmed and smear-positive TB. This resulted in wide CIs around the prevalence estimate (95% CI 1–6/1,000 for confirmed new smear-positive TB). Sampling bias due to resampling was minimized by replacing households that refused participation with generally similar neighboring households. For households that refused participation but provided some basic information on household members, no age or sex differences were found. For households that did not provide any information and refused participation, age or sex differences could not be tested.

Participants who were unable to provide a sputum specimen were considered to be smear negative and culture negative. This approach may have resulted in low prevalence estimates because some cases could have been missed. Okutan et al. (*21*) reported positive smears in 61% and positive cultures in 31% of gastric lavage specimens obtained from patients with suspected TB who were unable to provide sputum specimens. However, because the participants in our survey represented the entire population, not just those with suspected cases of TB, we anticipate that the proportion of missed cases will be considerably lower. The strength of our study was that sputum smear and culture was attempted for all participants. This differs from most surveys in which screening of symptoms, results of chest radiography, or both, are used as the basis for deciding which sputum specimen should be examined (*22*–*27*).

In conclusion, this prevalence survey supports the hypothesis that the prevalence of TB is extremely high in the area studied. Because previously treated smear-positive TB patients constituted more than half of the patients with prevalent smear-positive cases, the survey also suggests that these cases contribute to transmission of *M. tuberculosis*. Successful treatment of TB cases must be a priority in South Africa.
